# Evolutionary Tracing and Taxonomic Implications of the Mitochondrial Genome of *Gephyrocharax atracaudatus* (Meek and Hildebrand, 1912)

**DOI:** 10.3390/biology15090714

**Published:** 2026-04-30

**Authors:** Zhaowen Liu, Youkun Huang, Limin Yang, Jia Ye, Huiting Wu, Jiapan Pan, Chengtao Shan, Yudi Shan, Wenxi Wang, Junyi Wang, Zhuqing Feng, Siyu Chen

**Affiliations:** 1 School of Materials and Environmental Engineering, Chizhou University, Chizhou 247000, China; liuzhaowen92@163.com (Z.L.); yanglimin92@163.com (L.Y.);; 2 Anhui Provincial Key Laboratory for Quality and Safety of Agri-Products, School of Resource and Environment, Anhui Agricultural University, Hefei 230036, China

**Keywords:** *Gephyrocharax atracaudatus*, divergence time, evolutionary pressure, phylogenetic construction

## Abstract

*Gephyrocharax atracaudatus* is a rare freshwater fish in Panama. This study provides a comprehensive analysis of the mitochondrial genome of *G. atracaudatus*, highlighting its gene composition, codon usage, evolutionary pressures, and phylogenetic relationships. The findings reveal unique evolutionary patterns and divergence times of *G. atracaudatus* that enhance the understanding of the genetic diversity within Characiformes. The increasing availability of genomic data has prompted taxonomic revisions for numerous Characiformes species, encompassing corrections to species names, subfamily, and family classifications. This study reconfirmed the classification of *G. atracaudatus* as “*Gephyrocharax*, Stevardiinae, Stevardiidae, Characiformes”. A cross-analysis model for species differentiation and tracking was established using divergence time comparison and phylogenetic analysis. The results show that *G. atracaudatus*, *Hyphessobrycon roseus* and *Pristella maxillaris* converge on one branch, indicating that the genetic relationship may be the most similar, and it occurred approximately 90.45 Mya in the Cretaceous. This study establishes a robust model framework for understanding the evolution of *G. atracaudatus*, and correctly determines the biological classification status of *G. atracaudatus*, providing basic data support for clarifying the evolution mode of Characiformes.

## Simple Summary

*Gephyrocharax atracaudatus* originally belonged to “Characidae, Characiformes”. With the publication of more sequences, it seems that new insights have been gained regarding the classification of Characidae: the original Characidae should be divided into four new families (Spintheriobolidae, Stevardiidae, Characidae and Accentorhamphidae). This study jointly reveals the taxonomic status of *G. atracaudatus* through gene composition, codon usage characteristics, evolutionary pressure, and phylogenetic relationships. Concurrently, the evidence presented herein supports the categorisation of *G. atracaudatus* as part of the order Characiformes, specifically within the subfamily Stevardiinae and the family Stevardiidae (one of the four new families). This study establishes a robust model framework for understanding the evolution of *G. atracaudatus*, thereby providing fundamental data that supports the elucidation of the evolution mode of Characiformes.

## 1. Introduction

The mitochondrial genome (mitogenome) of vertebrates is compact and maternally inherited [1]. These features make it an excellent molecular marker for reconstructing phylogenetic relationships and estimating divergence times. High-throughput sequencing has rapidly increased the number of available whole mitochondrial genome sequences in recent years [2,3]. This expansion provides high-resolution data for understanding vertebrate evolutionary history across various taxonomic levels. Maternal inheritance and the high mutation rate of mitochondrial DNA supply essential data for reconstructing vertebrate lineages, identifying species, and defining conservation units [4]. When combined with phenotypic and ecological data, mitochondrial genomes can also indicate potential adaptive differentiation [5]. A typical vertebrate mitochondrial genome contains 13 protein-coding genes, two ribosomal RNA genes (12S rRNA and 16S rRNA), and 22 transfer RNA genes [6]. Detailed analysis of base composition bias and codon usage patterns in these sequences reveals the selective constraints and evolutionary dynamics acting upon them [7]. This approach helps identify adaptive changes that may occur under specific ecological conditions [8,9]. It thus provides valuable evidence for understanding how vertebrates respond to environmental changes. At the same time, integrating mitochondrial genome variation data from multiple species supports the assessment of genetic diversity [5]. This integration lays the groundwork for future incorporation of nuclear genome information and the construction of a more comprehensive phylogenetic framework.

*Gephyrocharax atracaudatus* (Meek and Hildebrand, 1912) represents a species that is emblematic of the genus *Gephyrocharax*. This species is found in the central-eastern region of Panama, where it occupies small, independent watersheds along both the Pacific and Caribbean coasts [10]. Its limited geographic range makes it an ideal system for investigating the formation and ecological differentiation of local freshwater fish species. The species is relatively small in body size and exhibits strong habitat specificity for particular microhabitats. These characteristics render it an accessible model for studying ecological interactions and evolutionary relationships among populations. Within its distribution range, *G. atracaudatus* frequently co-occurs with closely related species in the same ecological domain. This pattern reflects the potential role of watershed dynamics as a driving force in species formation [11]. The topography of Panama is characterized by high spatial heterogeneity and extensive water system isolation. These features collectively provide a natural experimental setting for examining the roles of geographic barriers and environmental factors in genetic differentiation [11].

In previous studies, *G. atracaudatus* was typically defined as belonging to Characidae [10]. However, the study have indicated that species assignments within Characidae may contain taxonomic errors [12]. Recent research using ultraconserved elements (UCEs) has revealed that *Gephyrocharax valenciae* is more closely related to *Corynopoma riisei* than to other *Gephyrocharax* species, including *Gephyrocharax venezuelae* and *Gephyrocharax machadoi* [13]. These species do not form a monophyletic group. This finding suggests that the conventional classification of *Gephyrocharax* may be problematic. The same study further proposes dividing the traditional Characidae into four distinct families: Spintheriobolidae, Stevardiidae, Characidae, and Accentorhamphidae. Under this new framework, *Gephyrocharax* is classified within Stevardiidae [13]. Because *G. atracaudatus* belongs to *Gephyrocharax*, its systematic position requires re-examination. Consequently, mitochondrial genome data for this species are urgently needed to verify its precise taxonomic placement within this revised familial framework.

The advent of molecular sequencing technology has enabled routine acquisition of complete mitochondrial genome data. This development has substantially enhanced the robustness of phylogenetic analysis. It overcomes the limitations of traditional single-gene fragments regarding node support and topological consistency [3]. Compared with morphological classification, genomic information provides higher resolution for reconstructing evolutionary relationships [2,14,15]. Single-gene fragments, such as *COX1* and *CytB*, have provided fundamental evidence for species identification and phylogenetic inference [16,17]. However, these markers contain limited information for analyzing deep temporal divergence or complex lineage relationships. At this point, multi-gene analyses covering the entire mitochondrial genome can provide more reliable phylogenetic signals. To address this gap, we conducted a second sequencing and systematic analysis of the complete mitochondrial genome of *G. atracaudatus*. We built upon the previous assembly [10]. For the first time, we estimated lineage divergence times based on this dataset. We also evaluated the systematic position of this species within Characidae. Compared with the costly and time-consuming process of de novo assembly of UCEs, mitochondrial sequencing offers a rapid and cost-effective approach for elucidating evolutionary relationships. This study provides new molecular evidence for reclassifying subfamilies within Characidae. It thus supplements the fragmented analyses of *G. atracaudatus* from previous studies. This work lays the foundation for subsequent biodiversity assessment and ecological research.

## 2. Materials and Methods

### 2.1. Sample Collection, DNA Extraction, PCR Amplification, and Sequencing

**Figure 1:**
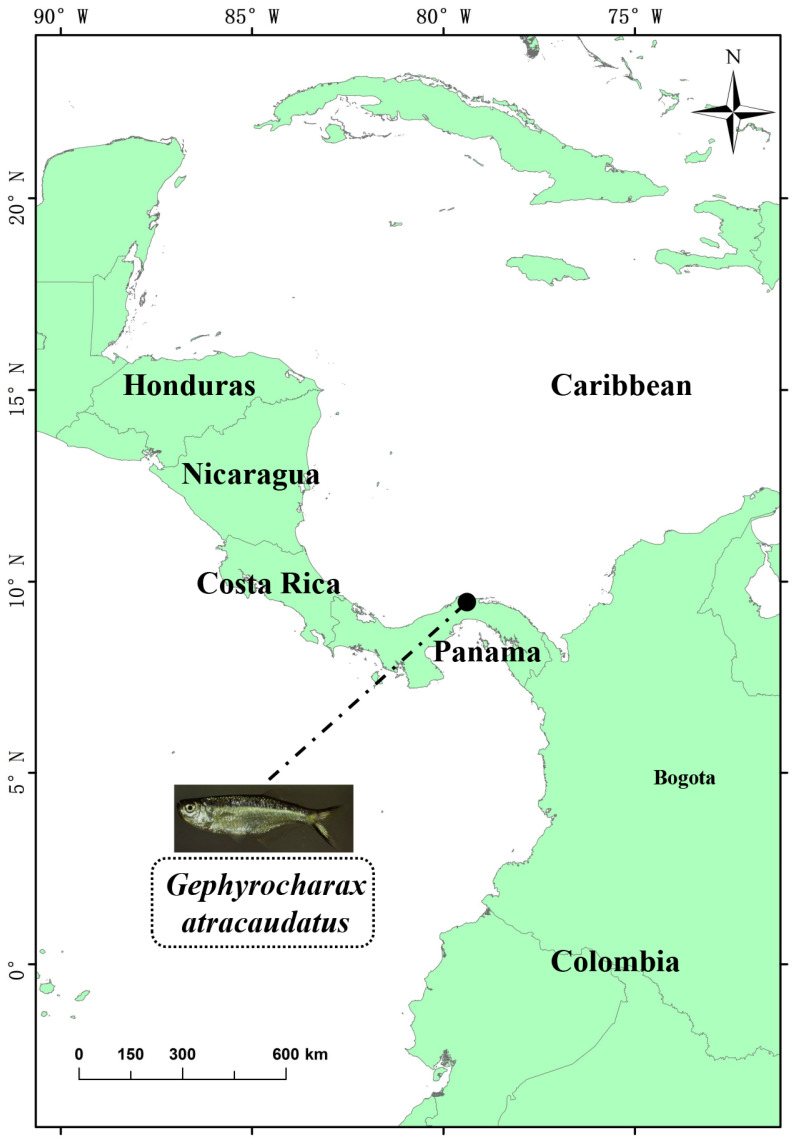
*G. atracaudatus*’ living water area and collection location.

Specimens of *G. atracaudatus* were collected from Flamenco Island, Panama (9°2′7″ N, 79°28′43″ W) on 16 August 2017 (Figure 1). One specimen (Carcass length: 33.6 mm, Female) was deposited in the Laboratory of the Museum of Materials and Environmental Engineering (Zhaowen Liu, liuzhaowen92@163.com) at Chizhou University, under voucher number GR2102145.

The experiment on the specimen was approved by the Animal Ethics Committee of Chizhou University (CZU-AEC-2017-06-04), and the experimental details were recorded in accordance with the ARRIVE 2.0 guidelines [18]. Following sample fixation, 20 mg of muscle tissue was obtained and total DNA was extracted using a modified phenol chloroform method with a 260/280 ratio ≥ 1.8 [3]. The complete mitochondrial genome was obtained through Illumina NovaSeq 6000 platform 150 bp double-ended sequencing. The high variability areas of the gap and control region were validated by Sanger sequencing [19]. The primer design was based on the mitochondrial genome of *G. atracaudatus* (MH636341) on NCBI (Supplementary Table S1), and the PCR system and cycling parameters were strictly followed in accordance with the instructions provided by specifications of the reagent kit (Takara, Beijing, China: PrimeSTAR^®^ Max DNA Polymerase).

### 2.2. Sequence Analysis, Assembly, and Mitochondrial Genome Annotation

The mitochondrial genome sequence was assembled in CodonCode Align 5.1.5 (CodonCode Corporation, Dedham, MA, USA) to obtain a complete circular DNA molecule. Subsequently, the MITOS online (http://mitos2.bioinf.uni-leipzig.de, accessed on 29 April 2026) server was employed for automatic annotation, based on the characteristics of metazoan mitochondria. Thereafter, a combination of the results of tRNAscan SE and further manual review and correction of the tRNA secondary structure predicted by MITOS was conducted (Supplementary Figure S1) [20].

### 2.3. Amino Acid Composition and Nucleotide Substitution Saturation Index of PCGs

The codon usage count and the relative synonymous codon usage (RSCU) value are calculated in MEGA 11 [21]. The non-synonymous mutation rate (Ka), synonymous mutation rate (Ks), and the Ka/Ks ratio of each PCG were estimated using DnaSP 5 with the Nei-Gojobori method (Jukes-Cantor correction) [22]. The nucleotide substitution saturation of PCG was evaluated in DAMBE 7: initially, the genetic distance is estimated using the TN93 model, and subsequently, a scatter plot is generated of the conversion and crossover on the genetic distance [23].

### 2.4. Relative Evolutionary Rate Analysis

We downloaded nucleotide sequences of 13 mitochondrial protein-coding genes from 20 species in NCBI (Table 1). We aligned each gene at the codon level using MUSCLE v3.8.31 (https://drive5.com/muscle/, accessed on 28 April 2026). We then concatenated the aligned sequences in the same species order using SequenceMatrix v1.7.8. This generated a 20 × 13 supermatrix. We implemented Tajima’s relative rate test using the TPCV module in the LINTRE (version 1) software package. We used the concatenated dataset for this analysis. We also evaluated significant rate heterogeneity in each branch [24].

### 2.5. Divergence Time Estimation

We estimated species divergence times in BEAST 2 using a strict molecular clock model. We implemented the following settings in the XML file. The nucleotide substitution model was GTR with four categories of discrete gamma distribution [25]. The tree prior was the Yule pure process (Yule Model). Log normal priors were set based on fossil records taken from the oldest credible fossil age of the corresponding node in the TimeTree database (http://www.timetree.org, accessed on 28 April 2026). The chain length was 10,000,000 generations, sampled once every 1000 generations, with the top 10% used as burn-in (MCMC). Convergence diagnosis was performed, ensuring that all parameters had an ESS greater than 200 with Tracer v1.7.2. Subsequently, the results were visualised using TVBOT to generate and plot a time-calibrated Maximum Credibility Tree (https://www.chiplot.online/tvbot.html, accessed on 28 April 2026) [26].

### 2.6. Phylogenetic Tree Construction

Twenty-one complete Characiformes mitochondrial genomes were downloaded from GenBank (https://www.ncbi.nlm.nih.gov/genbank/, accessed on 28 April 2026) for phylogenetic studies (Table 1). Two Saccopharyngiformes species, *Eurypharynx pelecanoides* and *Saccopharynx lavenbergi*, were used as outgroups. Excluding outgroups, we selected three families (Stevardiidae, Acestrorhamphidae and Bryconidae) of the order Characiformes for comparison (Table 1). The alignment of codon level nucleotide sequences of 13 mitochondrial protein coding genes (PCGs) was performed using MEGA 11 [21]. Subsequently, Gblocks 0.91b was utilised to eliminate segments exhibiting substandard alignment quality (allowing gap positions < 50% and minimum block length ≥ 10 bp) [27]. The reconstruction of phylogenetic relationships was conducted utilising Bayesian inference (BI) (Sample every 1000 generations and discard the first 25%, MrBayes 3.2.6) [28,29] and maximum likelihood (ML) (Node support is evaluated 1000 times) methodologies [30]. The resulting phylogenetic trees were visualized using FigTree v. 1.4.4 and its tools (https://itol.embl.de, accessed on 28 April 2026) [31].

## 3. Results

### 3.1. Characteristics, Structure and Overlapping of the Mitogenome

**Figure 2:**
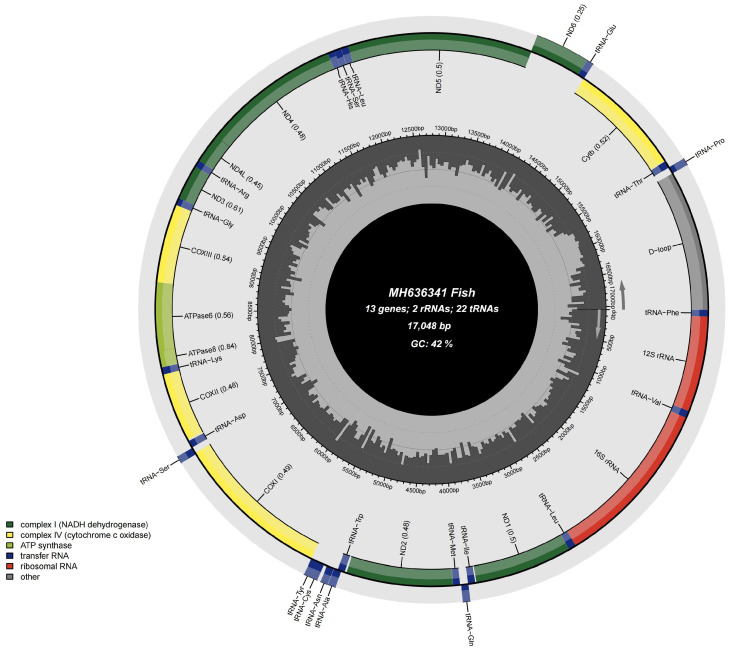
Gene map of the *G. atracaudatus* mitogenome.

The complete mitochondrial genome of *G. atracaudatus* spans 17,049 bp, as per GenBank accession MH636341 (Figure 2). The circular mitochondrial genome contained 13 protein-coding genes (PCGs), 2 ribosomal RNA genes (*12S rRNA* and *16S rRNA*), 22 transfer RNA genes, and a non-coding control region (D-loop). The L Strand hosted a smaller subset of genes, including 8 *tRNAs*: *tRNA-Gln*, *tRNA-Ala*, *tRNA-Asn*, *tRNA-Cys*, *tRNA-Tyr*, *tRNA-Ser*, *tRNA-Glu*, and *tRNA-Pro*. The remaining 14 *tRNAs* were positioned on the H strand. The *tRNA* genes ranged in length from 66 to 75 bp, reflecting their role in amino acid transfer during protein synthesis. Both the small (*12S rRNA*, 951 bp) and large (*16S rRNA*, 1683 bp) ribosomal subunit genes were present, with *tRNA-Phe* and *tRNA-Leu* positioned on the H strand, separated by *tRNA-Val* (Table 2). Compared with genes in other coding regions, ND6 exhibits unique and typical arrangement features on the mitochondrial genome, located on the L chain (Table 2) (Figure 2).

### 3.2. Protein-Coding Genes and Codon Usage and Mitogenome Mutations

**Figure 3:**
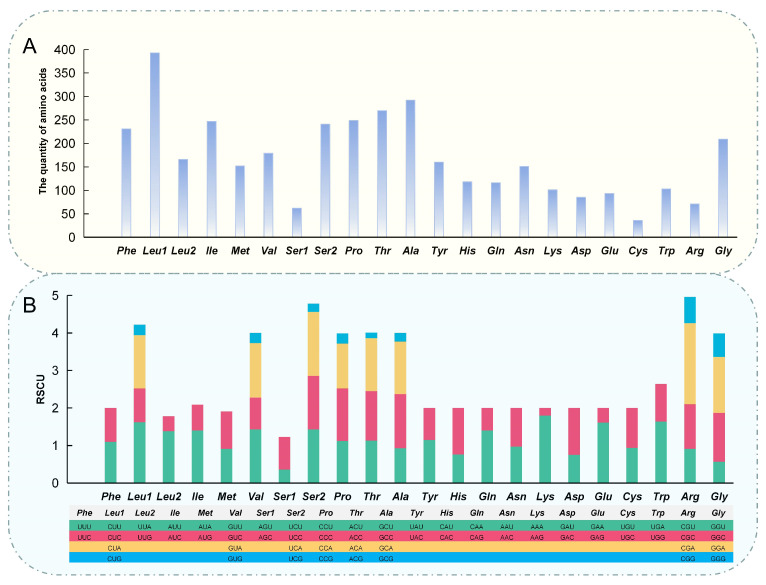
(**A**) Amino acid composition in the mitochondrial genome of *G. atracaudatus*. The *x*-axis and *y*-axis represent the amino acids and the number of occurrences of each amino acid in the 13 PCGs, respectively. (**B**) Relative synonymous codon usage (RSCU) in the mitochondrial genome of *G. atracaudatus*. The *y*-axis represents the frequency of codon usage for each amino acid in the 13 protein-coding genes (PCGs). Different colours indicate the different codons corresponding to each amino acid.

**Figure 4:**
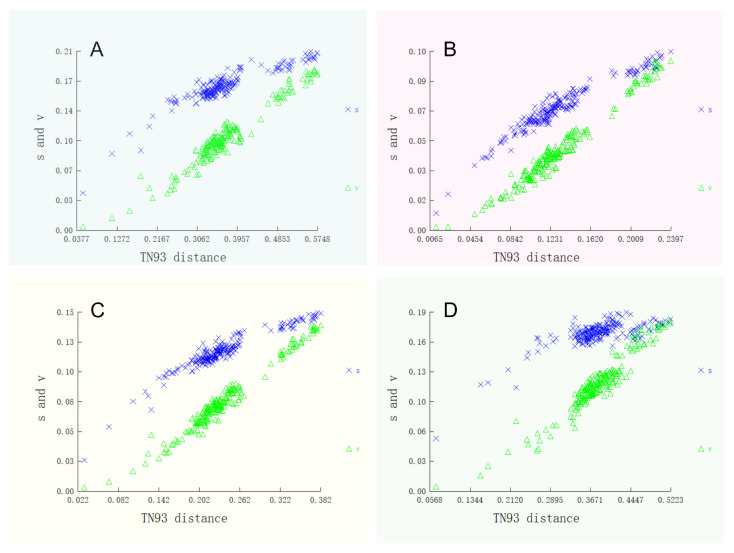
Nucleotide substitution saturation plots for all 13 protein-coding genes (PCGs) of Characiformes were shown as follows: (**A**) First codon positions; (**B**) second codon positions; (**C**) third codon positions; (**D**) first and second codon positions combined. In the plots, blue indicates transitions, while green represents transversions.

The coding region of the mitochondrial genome was 11,420 base pairs long, with 66.89% of it dedicated to PCGs (Table 2). GTG was the start codon of *COX1*, and other PCGs included ATG (ATG is a common start codon in the mitochondrial genome [5]). The Relative Synonymous Codon Usage (RSCU) analysis revealed notable variations in codon usage frequencies, highlighting evolutionary selection pressures on amino acids (Figure 3). *Leu1*, *Thr*, *Ala*, and *Gly* were more abundant, and the usage of four different codon types for *Leu1*, *Val*, *Ser2*, *Pro*, *Thr*, *Ala*, *Arg*, and *Gly* pointed to a buffering mechanism against genetic mutations (Figure 3). The TN93 (Tamura Nei, 1993) model is a commonly used nucleotide substitution model in molecular phylogenetics, which can simultaneously correct differences in nucleotide conversion rates and base frequency biases [32]. This study employed the TN93 model to analyze base substitution ratios and nucleotide frequencies across the three codon positions (Figure 4). Additionally, a network diagram was created to visualize correlations between gene fragments in the mitochondrial genome, including PCGs, *rRNAs*, and *tRNAs* (Supplementary Figure S1). Nodes represents individual gene fragments, while edges indicates significant correlations in sequence similarity or evolutionary rates.

### 3.3. Evolutionary Relationships in Stevardiidae Family

**Figure 5:**
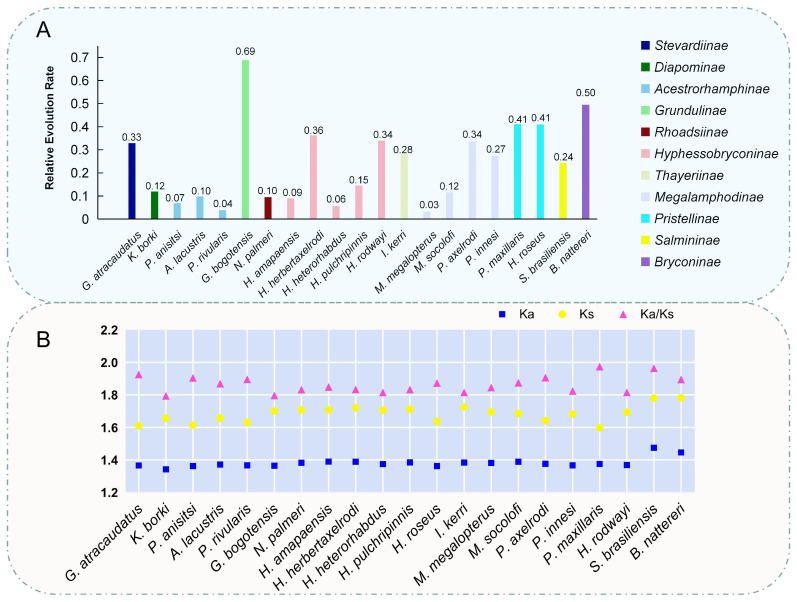
(**A**) Relative evolutionary pressure index of species based on the Characiformes mitochondrial genome model. Due to the minimal difference in evolutionary pressure index values between groups, we recalculated and optimized the data: y = lg (X + 1). (**B**) The ratio of nonsynonymous (Ka) to synonymous (Ks) substitutions (Ka/Ks), calculated using amino acids as data points, represents the mutational pressure index of the mitochondrial genome. Due to the close values of Ka, Ks, and Ka/Ks in some species, computational optimization was performed on the original data: y = e^x^.

In this study, we selected twenty representative mitogenomes of Stevardiidae to assess the evolutionary selection pressure on *G. atracaudatus*. Additionally, we included *Salminus brasiliensis* (*Salminus*, Salmininae, Characiformes) and *Brycon nattereri* (*Brycon*, Bryconinae, Characiformes), species with close phylogenetic relationships to *G. atracaudatus*, as well as *E. pelecanoides* and *S. lavenbergi*, which are less related, to validate the accuracy of our results through comparative analysis. Based on the gene sequences of 13 PCGs from the mitogenome, we assessed the relative evolutionary pressures across different species. The results (Figure 5A) revealed distinct pressure patterns among species from different families and genera. Compared to the evolutionary pressure faced by *G. atracaudatus*, the pressure on *Psalidodon anisitsi*, *Hyphessobrycon amapaensis*, *H. heterorhabdus*, *Megalamphodus megalopterus* and *P. rivularis* seem to be smaller. *Hyphessobrycon* contains a greater number and richness of species, with over 160 species discovered [33]. In contrast, *Grundulus bogotensis* and *B. nattereri* were under greater relative evolutionary pressure, Species with comparable evolutionary pressure indices to that of *G. atracaudatus* include *Hemigrammus rodwayi*, *H. herbertaxelrodi* and *Paracheirodon axelrodi*. When analyzing synonymous mutations in amino acids, the Ka and Ks values for different species showed relatively similar Ka/Ks ratios (Figure 5B). In Figure 5B, the Ka values for most species were smaller than their respective Ks values, and these values varied proportionally. However, *G. atracaudatus* stood out with a higher Ka/Ks ratio, a pattern also observed in *P. axelrodi* and *P. rivularis*.

### 3.4. Divergence Time and Phylogenetic Analysis

**Figure 6:**
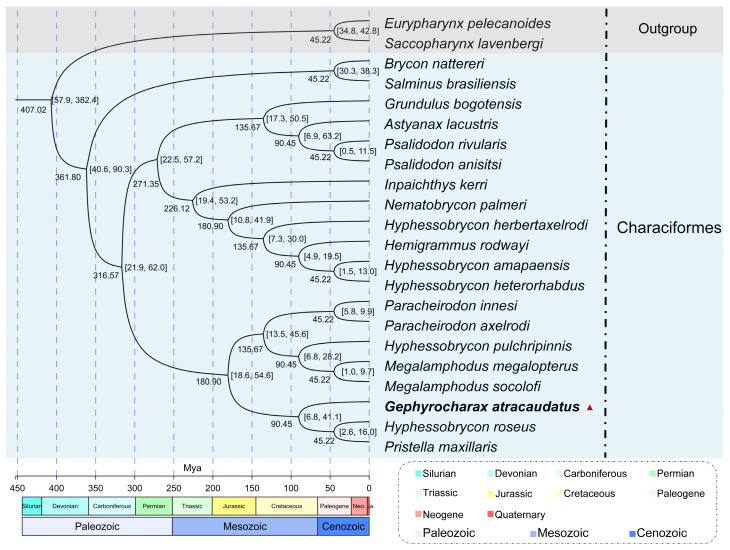
The divergence time and geological scale of the mitochondrial genomes of Characiformes species were illustrated in this figure. The evolutionary timeline was based on the relative divergence time between the outer groups *E. pelecanoides* and *S. lavenbergi*, with the divergence time between these species ranging from 118.3 to 25.9 million years ago (Mya). A divergence time of 39 Mya was chosen as the unit scale based on species affinity. The divergence time between *B. nattereri* and *S. brasiliensis* was estimated to be between 35.5 and 34.0 Mya, with 35 Mya chosen as the optimal unit scale, again based on species affinity (https//timetree.org, accessed on 28 April 2026).

**Figure 7:**
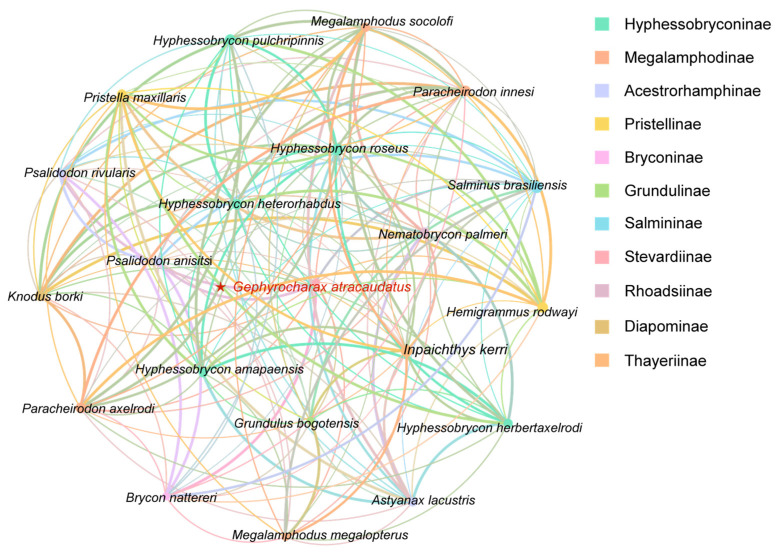
The interspecies correlation network diagram was constructed based on statistical analysis of mitochondrial genome data from 22 Characiformes species. These data, derived from Supplementary Table S2, include metrics such as ENC, CBI, SChi2, G + C2, G + C3s, G + Cc and G + C. Using Spearman correlation analysis (*p* < 0.05, r > 0.7), a symbiotic network was developed in R 4.4.0 software to explore correlations within Characiformes. The network was then visualized using Gephi 0.10.1.

**Figure 8:**
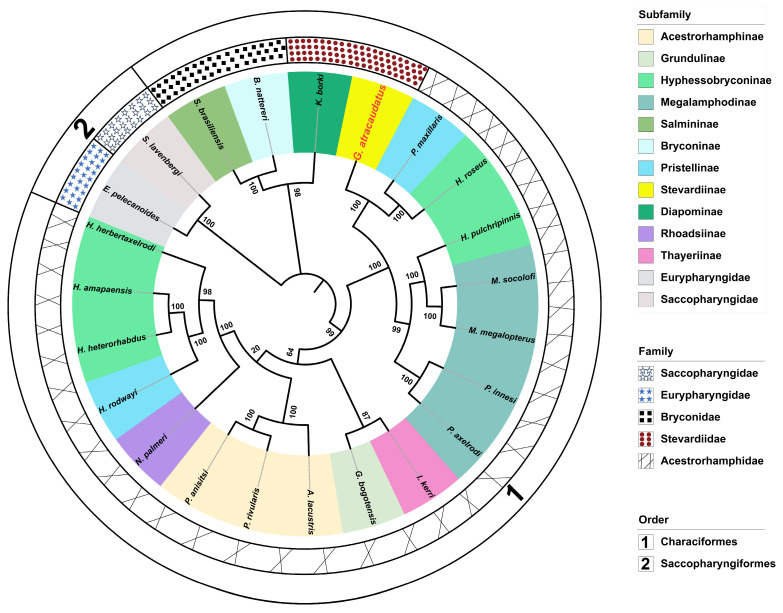
The phylogenetic tree of Characiformes was inferred from the nucleotide sequences of 13 protein-coding genes (PCGs) using both Bayesian inference (BI) and maximum likelihood (ML) methods. The numbers on the branches represent bootstrap values. These values indicate the confidence level of the phylogenetic relationships.

In this study, the PCGs of the mitochondrial genomes from twenty-two species were used to construct the evolutionary time tree (Figure 6). The relative differentiation times within the life evolution scale were calculated using the species pairs *E. pelecanoides* vs. *S. lavenbergi* and *B. nattereri* vs. *S. brasiliensis* (Supplementary Figure S2). *B. nattereri* and *S. brasiliensis*, as outgroups to Characiformes (Table 1), have a divergence time of 35.5–34.0 Mya. A divergence time of 35 Mya was selected as the optimal unit scale based on their species affinity (Supplementary Figure S2A). Similarly, *E. pelecanoides* and *S. lavenbergi*, as outgroups to Characiformes, have a divergence time ranging from 118.3–25.9 Mya. A divergence time of 39 Mya was chosen as the optimal unit scale for this pair, based on their species affinity (Supplementary Figure S2B). The results (Figure 6) (Table 1) suggested that the initial divergence between Saccopharyngiformes and Characiformes occurred approximately 361.80 Mya. In the divergence time topology, Characiformes species appeared to have split into two distinct clusters (Figure 6). In the first cluster, new species diverged approximately every 50–40 Mya, a pattern that seems consistent over time. Within the second clade, *G. atracaudatus* appeared to belong to an even older lineage. This species diverged from *Hyphessobrycon roseus* and *Pristella maxillaris* during an extensive differentiation period lasting approximately 90 million years. At the same time, an inter-species correlation network diagram was constructed based on statistical analysis of mitochondrial genome data from different species (Table 3) (Figure 7). As shown in Figure 7, nodes representing species within the same genus exhibited closer correlations, supporting the phylogenetic relationship and the relationship between evolutionary scales within the Characiformes population, a pattern that aligns with the results in Figure 6. All species, except for those in the cross-cluster (*Inpaichthys kerri* and *G. bogotensis*), showed consistency in both the time of divergence and in the construction of the sequence tree (Figure 6 and Figure 8). The tree structures from the two analyses displayed nearly identical clustering topologies. Notably, species within the same Subfamily often clustered with those from other Subfamily. Most species of Characiformes formed natural clusters, with *H. roseus* being a notable exception. The topology of the divergence times, a pattern also observed in the results of the systematic evolution, indicated that most of the new time points appeared during the Mesozoic period (Figure 8).

## 4. Discussion

Mitochondrial genomes contain abundant genetic information and serve as powerful molecular markers for establishing phylogenetic relationships and estimating divergence times [3,4]. In this study, *G. atracaudatus* showed a highly conserved gene arrangement (Figure 2). The use of GTG rather than the traditional ATG as the start codon for *COX1* indicates evolutionary flexibility. This variation may reflect adaptations that influence protein synthesis efficiency or mitochondrial function stability [5]. Incomplete stop codons are common in vertebrate mitochondrial genomes. These truncated codons are converted into complete termination signals through mRNA polyadenylation after transcription. Their widespread occurrence reflects strong selection for maintaining a compact genome size and replication efficiency [34]. Complete stop codons such as TAG and TAA also occur and serve as canonical termination signals without post-transcriptional modification. In many fish mitochondrial genomes, both types coexist, reflecting the dual influence of structural constraints and translational efficiency [3].

Codon usage patterns are key to understanding molecular evolution [35]. Amino acids with longer side chains tend to have more codon variants, reflecting their complex structural requirements (Figure 3) [36]. The abundance of *Leu1*, *Thr*, *Ala*, and *Gly* in this study suggests their critical role in protein synthesis for *G. atracaudatus*. The use of multiple codons for these amino acids points to a buffering mechanism against genetic mutations (Figure 3). This redundancy allows the genetic code to tolerate base changes without affecting protein function, contributing to genetic stability [37]. Conversely, codons with lower frequency for certain amino acids indicate vulnerability to mutations or drift, potentially leading to changes in codon preferences over time [36].

The TN93 model is commonly used in molecular phylogenetics. It corrects differences in nucleotide substitution rates and base frequency biases [32]. In this study, mutations at different codon positions affected amino acid evolution differently (Figure 4). Non-synonymous mutations at the second codon position significantly impact protein structure, while synonymous mutations at the third position have more subtle effects on genetic variation without altering amino acid sequences (Figure 4B,C) [38,39]. This distinction is crucial for understanding how genetic variations influence evolution. The larger impact of second-position mutations aligns with their critical role in determining amino acid properties [40]. The conservative nature of third-position mutations reflects evolutionary mechanisms that buffer against detrimental changes. The similarity between first and third codon frequency distributions indicates evolutionary conservatism in *G. atracaudatus*, where most mutations tend to be neutral. This aligns with the theory of purifying selection, where natural selection eliminates deleterious mutations [41].

A network diagram visualized correlations between mitochondrial gene fragments, including PCGs, rRNAs, and tRNAs (Supplementary Figure S1). Nodes represent individual gene fragments, while edges indicate significant correlations in sequence similarity or evolutionary rates. Strong correlations suggest that mitochondrial genes are conserved and co-evolve, likely driven by shared selective pressures and functional interactions [42]. Highly connected nodes indicate that these genes face shared selective pressures, possibly driven by the need for coordinated functionality in energy production and metabolic processes [43]. Stronger correlations may indicate shared selection pressures and evolutionary rates, suggesting that co-evolution is driven by functional interactions [44].

In this study, we selected 20 representative mitogenomes of Stevardiidae to assess the evolutionary selection pressure on *G. atracaudatus*. We evaluated the relative evolutionary pressure index based on the gene sequences of 13 PCGs from the mitochondrial genome. We compared pattern differences among species (Figure 5A). These species inhabit diverse environments. They may experience relatively lower mutation pressures, potentially due to smaller fluctuations in temperature or light. Compared with *Hyphessobrycon*, *G. atracaudatus* faced higher relative evolutionary pressure. This difference could be attributed to its specific habitat. *G. atracaudatus* is native to Flamenco Island, Panama (9°2′7″ N, 79°28′43″ E). It may be significantly influenced by interactions with other species or by landform evolution. These factors could contribute to greater evolutionary pressures on this species. In contrast, *Grundulus bogotensis* and *Brycon nattereri* were under greater relative evolutionary pressure. The specific factors influencing these pressures require further investigation, particularly regarding potential changes in their habitats. Species with comparable evolutionary pressure indices to that of *G. atracaudatus* include *Hemigrammus rodwayi*, *Hyphessobrycon herbertaxelrodi*, and *Paracheirodon axelrodi*. This similarity may be linked to the genetic stability of their mitochondrial genomes [45]. The consistent mutation pressure index in the mitochondrial genome confirms strict maternal inheritance characteristics. Combined with recorded coastal environmental changes, this consistency indicates that a common selection mechanism serves as an effective driving force for species distribution [46]. In amino acid synonymous mutation analysis (Figure 5B), the small variation in Ka values among different Characiformes species suggests that the frequency of neutral evolution was comparable among species. The accumulation of neutral mutations potentially contributed to the lack of environmental selectivity in their mitogenomes [47]. Compared with other species, *G. atracaudatus* showed a higher Ka/Ks ratio. This pattern may reflect the unique evolutionary dynamics of the species. The evolutionary selection pressure in *P. axelrodi* was similar to that in *G. atracaudatus* regarding the Ka/Ks ratio (Figure 5A). This similarity may be due to comparable habitat conditions. The habitat environments of *G. atracaudatus* (9°2′7″ N, 79°28′43″ E) and *P. axelrodi* (3°8′7″ N, 65°5′9″ E) are very similar. These localities are recorded in the Global Biodiversity Information Facility (GBIF) (https://www.gbif.org/species/2353911, accessed on 28 April 2026). This similarity suggests that the external environments and natural pressures faced by these species may follow comparable trends. For other Characiformes species, both constrained and divergent evolution appeared to be closely linked to gene mutations (Ka/Ks). Generally, evolutionary selection of mitogenomes tends to eliminate harmful mutations. This process preserves the stability of amino acid sequences over time [48].

Divergence time estimation has gained significant attention in evolutionary biology in recent years [49]. Advances in methodology and empirical research now allow for more accurate time tree estimations than ever before [29]. The molecular clock theory represents a key method for assessing genetic differences between species or populations [50,51]. It assumes that gene mutations accumulate at a relatively constant rate over time within a given lineage. This assumption allows scientists to estimate divergence times between species based on genetic variations [52]. This principle extends to biomolecules such as DNA and proteins, where mutations are presumed to occur at a predictable rate throughout evolution [53]. If mutation accumulation on an evolutionary branch is proportional to the duration of its independent evolutionary history, the substitution rate is expected to remain roughly constant [54,55]. This suggests that the mutation rate tends to stabilise over evolutionary time [51]. Molecular clocks often rely on specific genetic markers, such as mitochondrial DNA, ribosomal RNA genes, or certain protein-coding genes [56]. These markers are considered neutral because they are less influenced by selection pressures. They provide a more stable rate of evolution compared with other genomic regions [57]. By tracking the frequency of these mutations, we can estimate the time since two species or populations shared a common ancestor [53,57]. However, mutation rates are not always constant in practice. Factors such as selection pressure, environmental influences, and genetic drift can cause significant variation in mutation rates. This variation leads to deviations from the expected molecular clock [58,59]. Despite these challenges, molecular clocks remain a valuable tool in evolutionary biology. They offer important insights into the timing of evolutionary events.

In this study, the temporal divergence tree showed that the initial split between Saccopharyngiformes (outgroup) and Characiformes occurred approximately 361.8 million years ago. The families Acestrorhamphidae and Stevardiidae appeared approximately 316.5 million years ago, according to mitochondrial genome analysis (Figure 6) (Table 1). In the divergence time topology, Characiformes species split into two distinct clusters (Figure 6). In the first cluster, new species diverged approximately every 40 to 50 million years. This pattern appears consistent over time and may reflect the natural dynamics of species evolution. Environmental factors such as latitude, light, and temperature likely influenced convergent evolution and drove divergent evolutionary processes between regions [45]. However, comparison of mitochondrial genomes alone is insufficient to fully explain this phenomenon. A comprehensive analysis of the distribution ranges of all species worldwide is essential. Within the second clade, *G. atracaudatus* belonged to an older lineage. This species diverged from *Hyphessobrycon roseus* and *Pristella maxillaris* during an extensive differentiation period lasting approximately 90 million years. This result and the new classification are mutually corroborative. They support the recognition of Stevardiidae as sister to Acestrorhynchidae [12,13]. However, the temporal divergence tree constructed from the mitochondrial genome suggests that Stevardiidae evolved from Acestrorhamphidae. Therefore, more evidence is needed to validate Stevardiidae as an independent family from Acestrorhamphidae.

In the interspecies network diagram (Table 3) (Figure 7), different groups or isolated nodes may represent distinct evolutionary paths. These paths may be shaped by different selection pressures, mutation hotspots, or environmental factors. The nodes of species within the same genus show closer correlation (Figure 7). This pattern supports the relationship between phylogenetic relationships and evolutionary scales in Characiformes (Figure 6). These insights provide a foundation for further research into mitochondrial dysfunctions, evolutionary biology, and species-specific adaptations [6,26,54]. Combining phylogenetic or structural analyses with network data could provide a deeper understanding of mitochondrial genome evolution.

The phylogenetic tree results (Figure 8) were basically consistent with the temporal divergence topology (Figure 6). Both analyses showed the same clustering pattern. Generally, species of the same family and genus cluster together. However, species within the same subfamily often cluster with other subfamilies. This pattern seems related to the limited information content of mitochondrial genomes [19]. It is insufficient to construct trees using only part of the genome. Real divergence between species requires consideration of whether habitat, environmental temperature, or light have changed. The topology of divergence times indicated that most new divergence events appeared during the Mesozoic period (Figure 8). This pattern was also observed in the systematic evolution results. Glacial and crustal shifts likely altered original habitats. These changes may have led to geographical isolation within the same water systems. However, the ancient nature of mitochondrial genomes can result in discrepancies between gene trees and species trees. A more comprehensive analysis incorporating coastal geological changes would provide a clearer understanding of these patterns.

## 5. Conclusions

This study provides a comprehensive mitochondrial genome analysis of *G. atracaudatus*. It offers new insights into its molecular characteristics, evolutionary pressures, and phylogenetic position within Characiformes. The mitochondrial genome of *G. atracaudatus* exhibits a highly conserved gene arrangement. This pattern reinforces its suitability as a reliable marker for evolutionary inference. As more genomes have become publicly available, the species names, former names, and subfamily classifications of many Characiformes species have been corrected. Even family-level classifications have been revised [12,13]. Despite the publication of the complete mitochondrial genome of *G. atracaudatus*, further analysis is required to clarify its species differentiation and evolution. This study reconfirmed the classification of *G. atracaudatus* as *Gephyrocharax*, Stevardiinae, Stevardiidae, Characiformes. It also provided a solid theoretical basis for this classification. Nevertheless, this study has limitations. All phylogenetic and temporal inferences rely solely on mitochondrial genomes. These genomes represent a single, maternally inherited genetic system. This constraint may lead to discrepancies between gene trees and true species histories, particularly in ancient lineages. In addition, environmental interpretations remain indirect. Detailed ecological and geological data were not explicitly integrated. Consequently, while this work establishes a robust mitochondrial framework for understanding *G. atracaudatus* evolution, future studies are essential. These studies should incorporate nuclear genomes, broader taxon sampling, and environmental context to fully resolve its evolutionary history.

## Figures and Tables

**Table 1:** Species attribution and accession number.

Species	Former Name	Subfamily	Family	Order	Accession No.
*Gephyrocharax atracaudatus*	*\*	Stevardiinae	Stevardiidae	Characiformes	NC_042882
*Knodus borki*	*\*	Diapominae			NC_070390
*Psalidodon anisitsi*	*Hyphessobrycon anisitsi*	Acestrorhamphinae	Acestrorhamphidae		NC_066994
*Astyanax lacustris*	*\*				NC_053756
*Psalidodon rivularis*	*\*				NC_053757
*Grundulus bogotensis*	*\*	Grundulinae			NC_026195
*Nematobrycon palmeri*	*\*	Rhoadsiinae			NC_051983
*Hyphessobrycon amapaensis*	*\*	Hyphessobryconinae			NC_066989
*Hyphessobrycon herbertaxelrodi*	*\*				NC_050876
*Hyphessobrycon heterorhabdus*	*\*				NC_080887
*Hyphessobrycon pulchripinnis*	*\*				MW 331227
*Hyphessobrycon roseus*	*\*				MW 315749
*Inpaichthys kerri*	*\*	Thayeriinae			NC_057167
*Megalamphodus megalopterus*	*Hyphessobrycon megalopterus*	Megalamphodinae			NC_053878
*Megalamphodus socolofi*	*Hyphessobrycon socolofi*				NC_066990
*Paracheirodon axelrodi*	*\*				AB 898197
*Paracheirodon innesi*	*\*				KT 783482
*Pristella maxillaris*	*\*	Pristellinae			NC_066992
*Hemigrammus rodwayi*	*Hemigrammus armstrongi*				NC_066991
*Salminus brasiliensis*	*\*	Salmininae	Bryconidae		NC_024941
*Brycon nattereri*	*\*	Bryconinae			NC_051927
*Eurypharynx pelecanoides*	*\*	Eurypharynx	Eurypharyngidae	Saccopharyngiformes	AB 046473
*Saccopharynx lavenbergi*	*\*	Saccopharynx	Saccopharyngidae		AB 047825

**Table 2:** Features of the mitochondrial genomes of *G. atracaudatus*.

Mitogenome	Position		Length	Amino	Start/Stop	Intergenic Region (bp) *	Strand ^#^
	From/To		(bp)	Acid	Codon		Heavy/Light
*tRNA-Phe (F)*	1	68	68			0	H
*12S RNA*	69	1019	951			0	H
*tRNA-Val (V)*	1020	1091	72			0	H
*16S RNA*	1092	2774	1683			0	H
*tRNA-Leu^UUA^ (L1)*	2775	2849	75			0	H
*ND1*	2850	3821	972	324	ATG/TAA	0	H
*tRNA-Ile (I)*	3833	3904	72			11	H
*tRNA-Gln (Q)*	3973	3903	71			−2	L
*tRNA-Met (M)*	3984	4054	71			10	H
*ND2*	4056	5114	1059	353	ATG/TAG	1	H
*tRNA-Trp (W)*	5133	5205	73			18	H
*tRNA-Ala (A)*	5243	5168	76			34	L
*tRNA-Asn (N)*	5317	5246	72			2	L
*tRNA-Cys (C)*	5414	5349	66			32	L
*tRNA-Tyr (Y)*	5484	5414	71			−1	L
*COX1*	5486	7045	1560	520	GTG/AGG	1	H
*tRNA-Ser^UCA^ (S1)*	7104	7033	72			−13	L
*tRNA-Asp (D)*	7108	7179	72			3	H
*COX2*	7193	7880	688	229	ATG/T	13	H
*tRNA-Lys (K)*	7887	7953	67			6	H
*ATP8*	7955	8122	168	56	ATG/TAG	1	H
*ATP6*	8113	8795	683	227	ATG/TA	−10	H
*COX3*	8795	9578	784	261	ATG/T	−1	H
*tRNA-Gly (G)*	9579	9651	73			0	H
*ND3*	9652	10000	349	116	ATG/T	0	H
*tRNA-Arg (R)*	10001	10069	69			0	H
*ND4L*	10070	10366	297	99	ATG/TAA	0	H
*ND4*	10360	11740	1381	460	ATG/T	−7	H
*tRNA-His (H)*	11741	11809	69			0	H
*tRNA-Ser^AGC^ (S2)*	11810	11877	68			0	H
*tRNA-Leu^CUA^ (L2)*	11879	11951	73			1	H
*ND5*	11952	13787	1836	617	ATG/TAA	0	H
*ND6*	14299	13784	516	171	ATG/TAG	−4	L
*tRNA-Glu (E)*	14367	14300	68			0	L
*Cyt b*	14371	15507	1137	379	ATG/TAA	3	H
*tRNA-Thr (T)*	15512	15585	74			4	H
*tRNA-Pro (P)*	15652	15582	71			−4	L
Dloop	15653	17049	1397			0	H

* Intergenic region: non-coding bases between the feature on the same line and the line below, with a negative number indicating an overlap. ^#^ H: heavy strand; L: light strand.

**Table 3:** The sequence characteristics and statistical analysis of 13 protein-coding genes (PCGs), 22 transfer RNAs (*tRNAs*) and 2 ribosomal RNAs (*rRNAs*) in the mitochondrial genome of *G. atracaudatus* were investigated. The analysis was performed using gene sequences from the mitochondrial genome, which were processed using DnaSP v6 software.

Gene	G + C Content	Total Number of Mutations	Nucleotide Diversity (ND)	ND’s Standard Deviation	Average Number of Nucleotide Differences	Number of Haplotypes	Haplotype Diversity	Standard Deviation of Haplotype Diversity	Fu’s Fs Statistic	Tajima’s D	Number of Segregating Sites Analyzed	Fu and Li’s D-Star Test Statistic	Fu and Li’s F-Star Test Statistic	Achaz Y-Star Test Statistic
*tRNA-Phe (F)*	0.41	98	0.31	0.051	21.4	19	0.99	0.018	−3.6	−0.874	22	−0.145	−0.06	0.371
*tRNA-Val (V)*	0.5	28	0.08	0.008	5.7	17	0.98	0.02	−8.47	−1.03	20	−0.316	−0.521	−1.092
*tRNA-Leu^UUA^ (L1)*	0.51	85	0.26	0.038	19.6	19	0.99	0.018	−4.01	−0.696	33	−0.413	−0.424	−0.156
*tRNA-Ile (I)*	0.51	68	0.25	0.052	18	20	0.99	0.016	−6.22	−0.188	41	1.567	1.275	−0.701
*tRNA-Gln (Q)*	0.39	67	0.22	0.031	15.9	17	0.98	0.02	−2.42	−0.578	39	−1.14	−0.983	0.608
*tRNA-Met (M)*	0.4	115	0.36	0.05	25.2	17	0.99	0.021	−2.06	−0.979	28	−0.105	−0.2	−0.491
*tRNA-Trp (W)*	0.4	133	0.45	0.033	31	20	0.99	0.016	−3.56	−0.664	13	0.311	0.177	−0.529
*tRNA-Ala (A)*	0.38	36	0.1	0.014	7	18	0.99	0.019	−8.79	−1.178	22	−1.146	−1.263	−1.067
*tRNA-Asn (N)*	0.48	38	0.09	0.018	6.7	17	0.98	0.02	−7.28	−1.448	27	−2.323	−2.267	−0.547
*tRNA-Cys (C)*	0.49	127	0.46	0.038	30.1	20	0.99	0.016	−3.68	−0.601	12	0.627	0.615	0.053
*tRNA-Tyr (Y)*	0.49	67	0.21	0.045	14.8	19	0.99	0.018	−5.47	−0.822	33	0.284	0.039	−0.978
*tRNA-Ser^UCA^ (S1)*	0.48	60	0.14	0.059	10.1	10	0.89	0.039	1.77	−1.58	36	0.726	0.119	−2.195
*tRNA-Asp (D)*	0.36	116	0.44	0.042	31.3	18	0.98	0.019	−1.04	−0.116	19	1.008	1.113	0.559
*tRNA-Lys (K)*	0.5	107	0.25	0.067	18.2	17	0.98	0.02	−1.9	−1.577	26	0.127	−0.138	−1.143
*tRNA-Gly (G)*	0.3	101	0.4	0.037	28	17	0.98	0.02	−0.47	−0.007	24	0.465	0.522	0.324
*tRNA-Arg (R)*	0.41	74	0.28	0.043	19.4	19	0.99	0.018	−4.04	−0.22	36	0.565	0.601	0.251
*tRNA-His (H)*	0.31	75	0.19	0.046	13.4	17	0.98	0.02	−3.16	−1.438	32	0.061	−0.317	−1.667
*tRNA-Ser^AGC^ (S2)*	0.48	97	0.23	0.058	15.6	18	0.98	0.019	−3.69	−1.713	30	−1.947	−2.055	−1.684
*tRNA-Leu^CUA^ (L2)*	0.42	50	0.09	0.042	6.7	12	0.89	0.059	1.28	−2.067	36	−3.139	−3.042	−0.961
*tRNA-Glu (E)*	0.42	65	0.22	0.05	15.2	17	0.97	0.026	−2.61	−0.631	34	0.835	0.64	−0.582
*tRNA-Thr (T)*	0.49	91	0.35	0.031	25.4	20	0.99	0.0003	−4.41	0.02	36	−0.563	−0.405	0.673
*tRNA-Pro (P)*	0.36	86	0.26	0.053	18.4	19	0.99	0.018	−4.31	−0.934	36	0.565	0.367	−0.673
*ND1*	0.42	1825	0.5	0.048	482	20	0.99	0.016	2.65	−0.207	296	1.495	1.584	0.563
*ND2*	0.41	2565	0.54	0.042	563.5	20	0.99	0.016	2.94	−0.871	59	1.141	1.08	−0.005
*ND3*	0.43	484	0.31	0.047	106.6	20	0.99	0.016	−0.14	−0.859	175	0.999	0.483	−1.652
*ND4L*	0.46	284	0.24	0.011	71.9	20	0.99	0.016	−0.99	−0.367	102	0.107	0.175	0.318
*ND4*	0.42	1850	0.28	0.033	382.4	20	0.99	0.016	2.23	−1.065	619	−2.405	−2.261	0.089
*ND5*	0.4	3525	0.35	0.061	642.6	20	0.99	0.016	3.18	−1.431	467	−0.456	−0.739	−1.493
*ND6*	0.42	939	0.35	0.042	180.4	20	0.99	0.016	0.87	−1.282	149	−1.564	−1.576	−0.655
*COX1*	0.44	1025	0.18	0.006	274.5	20	0.99	0.016	1.63	−0.152	350	0.992	1.116	0.637
*COX2*	0.42	1122	0.28	0.059	192.2	19	0.99	0.018	2.47	−1.593	270	−1.865	−1.94	−1.335
*COX3*	0.45	470	0.16	0.006	125.2	19	0.99	0.018	1.46	−0.173	190	0.573	0.632	0.347
*ATP8*	0.35	249	0.33	0.029	54.8	20	0.99	0.016	−1.68	−0.857	59	−0.079	−0.176	−0.455
*ATP6*	0.41	1306	0.39	0.051	264.4	20	0.99	0.016	1.57	−1.128	180	−0.12	−0.363	−1.119
*Cyt b*	0.42	1345	0.24	0.037	267.8	20	0.99	0.016	1.59	−1.178	559	−2.554	−2.394	0.175
*12S RNA*	0.46	2535	0.63	0.018	602.4	20	0.99	0.016	3.06	−0.603	50	−0.761	−0.798	−0.411
*16S RNA*	0.43	4446	0.64	0.019	1060.9	20	0.99	0.016	4.12	−0.588	55	0.766	0.639	−0.332

## Data Availability

The data that support the findings of this study are openly available in National Center for Biotechnology Information at https://www.ncbi.nlm.nih.gov/; reference number: https://www.ncbi.nlm.nih.gov/nuccore/MH636341.1/, accessed on 28 April 2026.

## Supplementary Materials

The following supporting information can be downloaded at: https://www.mdpi.com/article/10.3390/biology15090714/s1, Figure S1: The Gene Correlation Network Diagram; Figure S2: Corresponding Differentiation Time of Reference Unit Species; Table S1: PCR Amplification Steps, Table S2: The Sequence Characteristics of 13 Protein-Coding Genes (PCGs) of Characidae.

## References

[1] Abe K.T., Mariguela T.C., Avelino G.S., Foresti F., Oliveira C. (2014). Systematic and historical biogeography of the *Bryconidae* (*Ostariophysi*: *Characiformes*) suggesting a new rearrangement of its genera and an old origin of Mesoamerican ichthyofauna. BMC Evol. Biol..

[2] Al Arab M., zu Siederdissen C.H., Tout K., Sahyoun A.H., Stadler P.F., Bernt M. (2017). Accurate annotation of protein-coding genes in mitochondrial genomes. Mol. Phylogenet. Evol..

[3] Al Zahrani M.R., Al Ghamdi K.M., Aljameeli M.M., Bakr M.N., Alghamdi T.S., Haider T.A., Gharsan F.N., Alghamdi S.Q., Abdella E.M., Mahyoub J.A. (2023). Phylogenetic analysis of Aedes species distributed in Taif Province, Saudi Arabia, based on mitochondrial COX1 sequences. Entomol. Res..

[4] Arbogast B.S., Edwards S.V., Wakeley J., Beerli P., Slowinski J.B. (2002). Estimating divergence times from molecular data on phylogenetic and population genetic timescales. Annu. Rev. Ecol. Syst..

[5] Bannikova A.A., Lebedev V.S. (2022). The concept of modern molecular clock and experience in estimating divergence times of *Eulipotyphla* and *Rodentia*. Zhurnal Obs. Biol..

[6] Blokzijl F., de Ligt J., Jager M., Sasselli V., Roerink S., Sasaki N., Huch M., Boymans S., Kuijk E., Prins P. (2016). Tissue-specific mutation accumulation in human adult stem cells during life. Nature.

[7] Bouckaert R., Heled J., Kühnert D., Vaughan T., Wu C.-H., Xie D., Suchard M.A., Rambaut A., Drummond A.J. (2014). BEAST 2: A Software Platform for Bayesian Evolutionary Analysis. PLoS Comput. Biol..

[8] Bromham L., Duchêne S., Hua X., Ritchie A.M., Duchêne D.A., Ho S.Y.W. (2018). Bayesian molecular dating: Opening up the black box. Biol. Rev..

[9] Bulmer M. (1991). The selection-mutation-drift theory of synonymous codon usage. Genetics.

[10] Chen W.S., Wang H.Y. (2015). Variance estimation for nucleotide substitution models. Mol. Phylogenet. Evol..

[11] Cruz-Salazar B., George-Miranda S., Andraca-Gómez G. (2023). Analysis of the correlation between genetic and species diversity in a temperate forest: Variation in cohorts and effect of disturbance. Flora.

[12] De Giorgi C., Saccone C. (1989). Mitochondrial genome in animal cells. Structure organization, and evolution. Cell Biophys..

[13] Donath A., Jühling F., Al-Arab M., Bernhart S.H., Reinhardt F., Stadler P.F., Middendorf M., Bernt M. (2019). Improved annotation of protein-coding genes boundaries in metazoan mitochondrial genomes. Nucleic Acids Res..

[14] Drummond A.J., Suchard M.A., Xie D., Rambaut A. (2012). Bayesian Phylogenetics with BEAUti and the BEAST 1.7. Mol. Biol. Evol..

[15] Du Sert N.P., Ahluwalia A., Alam S., Avey M.T., Baker M., Browne W.J., Clark A., Cuthill I.C., Dirnagl U., Emerson M. (2020). Reporting animal research: Explanation and elaboration for the ARRIVE guidelines 2.0. PLoS Biol..

[16] Elías D.J., McMahan C.D., Alda F., García-Alzate C., Hart P.B., Chakrabarty P. (2023). Phylogenomics of trans-Andean tetras of the genus *Hyphessobrycon durbin* 1908 (*Stethaprioninae*: *Characidae*) and colonization patterns of Middle America. PLoS ONE.

[17] Fang Y., Zhang J., Wu R., Xue B., Qian Q., Gao B. (2018). Genetic Polymorphism Study on *Aedes albopictus* of Different Geographical Regions Based on DNA Barcoding. BioMed Res. Int..

[18] Feng P., Zhao H., Lu X. (2015). Evolution of mitochondrial DNA and its relation to basal metabolic rate. Mitochondrial DNA.

[19] Forest F. (2009). Calibrating the Tree of Life: Fossils, molecules and evolutionary timescales. Ann. Bot..

[20] Friedman J.R., Nunnari J. (2014). Mitochondrial form and function. Nature.

[21] Guindon S., Dufayard J.-F., Lefort V., Anisimova M., Hordijk W., Gascuel O. (2010). New Algorithms and Methods to Estimate Maximum-Likelihood Phylogenies: Assessing the Performance of PhyML 3.0. Syst. Biol..

[22] Huang Y.K., Liu B.J., Meng F., Wang Q., Zhu K.H., Zhang J.S., Jing F., Xia L., Liu Y.F. (2019). The complete mitochondrial genome of *Poecilia formosa* (*Poecilia*, *Cyprinodontidae*) and phylogenetic studies of cyprinodontiformes. Mitochondrial DNA Part B Resour..

[23] Huang Y.K., Liu B.J., Zhu K.H., Zhang J.S., Jing F., Xia L.P., Liu Y.F. (2019). The complete mitochondrial genome of *Gephyrocharax atracaudatus* (*Characiformes*, *Characidae*) and phylogenetic studies of Characiformes. Mitochondrial DNA Part B Resour..

[24] Huang Y.K., Zhu K.H., Yang Y.W., Fang L.C., Liu Z.W., Ye J., Jia C., Chen J., Jiang H. (2023). Comparative Analysis of Complete Mitochondrial Genome of *Ariosoma meeki* (Jordan and Snider, 1900), Revealing Gene Rearrangement and the Phylogenetic Relationships of Anguilliformes. Biology.

[25] Laird S., Jensen H.J. (2007). Correlation, selection and the evolution of species networks. Ecol. Model..

[26] Li X.J., Giorgi E.E., Marichannegowda M.H., Foley B., Xiao C., Kong X.-P., Chen Y., Gnanakaran S., Korber B., Gao F. (2020). Emergence of SARS-CoV-2 through recombination and strong purifying selection. Sci. Adv..

[27] Librado P., Rozas J. (2009). DnaSP v5: A software for comprehensive analysis of DNA polymorphism data. Bioinformatics.

[28] Liu D., Guo H., Zhu J., Qu K., Chen Y., Guo Y., Ding P., Yang H., Xu T., Jing Q. (2022). Complex Physical Structure of Complete Mitochondrial Genome of *Quercus acutissima* (Fagaceae): A Significant Energy Plant. Genes.

[29] Lü Z., Zhu K., Jiang H., Lu X., Liu B., Ye Y., Jiang L., Liu L., Gong L. (2019). Complete mitochondrial genome of *Ophichthus brevicaudatus* reveals novel gene order and phylogenetic relationships of Anguilliformes. Int. J. Biol. Macromol..

[30] Luo A., Ho S.Y.W. (2018). The molecular clock and evolutionary timescales. Biochem. Soc. Trans..

[31] Melo B.F., Ota R.P., Benine R.C., Carvalho F.R., Lima F.C.T., Mattox G.M.T., Souza C.S., Faria T.C., Reia L., Roxo F.F. (2024). Phylogenomics of Characidae, a hyper-diverse Neotropical freshwater fish lineage, with a phylogenetic classification including four families (*Teleostei*: *Characiformes*). Zool. J. Linn. Soc..

[32] Miya M., Takeshima H., Endo H., Ishiguro N.B., Inoue J.G., Mukai T., Satoh T.P., Yamaguchi M., Kawaguchi A., Mabuchi K. (2003). Major patterns of higher teleostean phylogenies: A new perspective based on 100 complete mitochondrial DNA sequences. Mol. Phylogenet. Evol..

[33] Monroe J.G., Srikant T., Carbonell-Bejerano P., Becker C., Lensink M., Exposito-Alonso M., Klein M., Hildebrandt J., Neumann M., Kliebenstein D. (2022). Mutation bias reflects natural selection in *Arabidopsis thaliana*. Nature.

[34] Nosek J., Tomáska L. (2003). Mitochondrial genome diversity:: Evolution of the molecular architecture and replication strategy. Curr. Genet..

[35] Oliveira M.T., Haukka J., Kaguni L.S. (2015). Evolution of the Metazoan Mitochondrial Replicase. Genome Biol. Evol..

[36] Peng Y., Yan H., Guo L., Deng C., Wang C., Wang Y., Kang L., Zhou P., Yu K., Dong X. (2022). Reference genome assemblies reveal the origin and evolution of allohexaploid oat. Nat. Genet..

[37] Pyron R.A. (2011). Divergence Time Estimation Using Fossils as Terminal Taxa and the Origins of *Lissamphibia*. Syst. Biol..

[38] Ray S.K., Baruah V.J., Satapathy S.S., Banerjee R. (2014). Cotranslational protein folding reveals the selective use of synonymous codons along the coding sequence of a low expression gene. J. Genet..

[39] Robinson J., Kyriazis C.C., Yuan S.C., Lohmueller K.E. (2023). Deleterious Variation in Natural Populations and Implications for Conservation Genetics. Annu. Rev. Anim. Biosci..

[40] Ronquist F., Klopfstein S., Vilhelmsen L., Schulmeister S., Murray D.L., Rasnitsyn A.P. (2012). A Total-Evidence Approach to Dating with Fossils, Applied to the Early Radiation of the Hymenoptera. Syst. Biol..

[41] Ronquist F., Teslenko M., van der Mark P., Ayres D.L., Darling A., Höhna S., Larget B., Liu L., Suchard M.A., Huelsenbeck J.P. (2012). MrBayes 3.2: Efficient Bayesian Phylogenetic Inference and Model Choice Across a Large Model Space. Syst. Biol..

[42] Rosenberg N.A., Nordborg M. (2002). Genealogical trees, coalescent theory and the analysis of genetic polymorphisms. Nat. Rev. Genet..

[43] Rusin M., Çetintaş O., Ghazali M., Sándor A.D., Yanchukov A. (2024). Underworld: Evolution of blind mole rats in Eastern Europe. Mamm. Biol..

[44] Shen X.K., Song S.L., Li C., Zhang J.Z. (2022). Synonymous mutations in representative yeast genes are mostly strongly non-neutral. Nature.

[45] Sitnikova T. (1996). Bootstrap method of interior-branch test for phylogenetic trees. Mol. Biol. Evol..

[46] Takezaki N., Rzhetsky A., Nei M. (1995). Phylogenetic test of the molecular clock and linearized trees. Mol. Biol. Evol..

[47] Talavera G., Castresana J. (2007). Improvement of phylogenies after removing divergent and ambiguously aligned blocks from protein sequence alignments. Syst. Biol..

[48] Tamura K., Nei M. (1993). Estimation of the number of nucleotide substitutions in the control region of mitochondrial DNA in humans and chimpanzees. Mol. Biol. Evol..

[49] Tamura K., Stecher G., Kumar S. (2021). MEGA11 Molecular Evolutionary Genetics Analysis Version 11. Mol. Biol. Evol..

[50] Tanner A.R., Fuchs D., Winkelmann I.E., Gilbert M.T.P., Pankey M.S., Ribeiro Â.M., Kocot K.M., Halanych K.M., Oakley T.H., da Fonseca R.R. (2017). Molecular clocks indicate turnover and diversification of modern coleoid cephalopods during the Mesozoic Marine Revolution. Proc. R. Soc. B Biol. Sci..

[51] Vanegas-Ríos J.A. (2016). Taxonomic review of the Neotropical genus *Gephyrocharax eigenmann*, 1912 (*Characiformes*, *Characidae*, *Stevardiinae*). Zootaxa.

[52] Wang M.T., Hou Z.Y., Li C., Yang J.P., Niu Z.T., Xue Q.Y., Liu W., Ding X.Y. (2023). Rapid structural evolution of Dendrobium mitogenomes and mito-nuclear phylogeny discordances in *Dendrobium* (*Orchidaceae*). J. Syst. Evol..

[53] Xia X., Xie Z. (2001). DAMBE: Software package for data analysis in molecular biology and evolution. J. Hered..

[54] Yang L.M., Xue J.F., Zhao X.M., Ding K., Liu Z.W., Wang Z.S.Y., Chen J.-B., Huang Y.K. (2024). Mitochondrial Genome Characteristics Reveal Evolution of *Acanthopsetta nadeshnyi* (Jordan and Starks, 1904) and Phylogenetic Relationships. Genes.

[55] Yang Y.Q., Wang J.J., Dai R.H., Wang X.Y. (2023). Structural Characteristics and Phylogenetic Analysis of the Mitochondrial Genomes of Four *Krisna* Species (*Hemiptera: Cicadellidae*: *Iassinae*). Genes.

[56] Yi S.V. (2007). Understanding neutral genomic molecular clocks. Evol. Biol..

[57] Yuan L.L., Liu H.Y., Ge X.Y., Yang G.Y., Xie G.L., Yang Y.X. (2022). A Mitochondrial Genome Phylogeny of *Cleridae* (*Coleoptera*, *Cleroidea*). Insects.

[58] Zhu T., Sato Y., Sado T., Miya M., Iwasaki W. (2023). MitoFish, MitoAnnotator, and MiFish Pipeline: Updates in 10 Years. Mol. Biol. Evol..

[59] Zuckerkandl E., Pauling L. (1965). Molecules as documents of evolutionary history. J. Theor. Biol..

