# Supplementary material for: Evolutionary Tracing and Taxonomic Implications of the Mitochondrial Genome of Gephyrocharax atracaudatus (Meek and Hildebrand, 1912)
Source: Biology (Basel). 2026 Apr 30;15(9):714. doi: 10.3390/biology15090714 (PMC13162575; doi:10.3390/biology15090714)
Supplement: Supplementary file 1 [file biology-15-00714-s001.zip › biology-4177314-supplementary.pdf]

Supplementary Table S1. PCR amplification steps.

	Reagent	Amount of Usage	the Final Concentration
Preparation of PCR reaction solution (Total 50 μ L)	PrimeSTAR Max Premix（2X）	25 μl	1 X
	Primer 1	15 pmol	0.3 μM
	Primer 2	15 pmol	0.3 μM
	**Temperature**	**Time**	**Cycles**
PCR reaction conditions	98 ℃	10 sec	
	55 ℃	60 sec/kb	35 Cycles
	72 ℃	60 sec/kb	

Supplementary Table S2. The sequence characteristics of 13 protein-coding genes (PCGs) of Characidae

Species	ENC	CBI	SChi2	G+C2	G+C3s	G+Cc	G+C
A. lacustris	57.417	0.136	0.079	0.431	0.426	0.436	0.421
B. nattereri	57.245	0.139	0.077	0.438	0.419	0.437	0.422
G. atracaudatus	56.435	0.163	0.092	0.436	0.415	0.431	0.417
G. bogotensis	55.454	0.203	0.126	0.405	0.371	0.409	0.398
H. armstrongi	56.372	0.172	0.099	0.438	0.423	0.435	0.419
H. amapaensis	56.491	0.174	0.099	0.422	0.411	0.423	0.409
H. anisitsi	56.616	0.149	0.09	0.447	0.426	0.441	0.427
H. herbertaxelrodi	56.759	0.164	0.095	0.42	0.413	0.423	0.408
H. heterorhabdus	56.581	0.166	0.095	0.437	0.42	0.432	0.418
H. megalopterus	56.183	0.176	0.1	0.412	0.399	0.413	0.401
H. pulchripinnis	57.954	0.122	0.076	0.455	0.44	0.453	0.438
H. roseus	57.145	0.149	0.085	0.452	0.439	0.452	0.437
H. socolofi	56.891	0.165	0.087	0.428	0.419	0.43	0.417
I. kerri	55.607	0.201	0.118	0.396	0.389	0.401	0.389
K. borki	55.401	0.173	0.11	0.431	0.418	0.43	0.416
N. palmeri	56.074	0.174	0.105	0.397	0.393	0.402	0.389
P. axelrodi	56.322	0.178	0.101	0.428	0.416	0.425	0.413
P. innesi	56.331	0.176	0.102	0.436	0.417	0.429	0.415
P. maxillaris	57.06	0.147	0.085	0.439	0.433	0.442	0.427
P. rivularis	57.088	0.14	0.083	0.453	0.429	0.445	0.431
S. brasiliensis	58.075	0.13	0.071	0.464	0.446	0.464	0.452

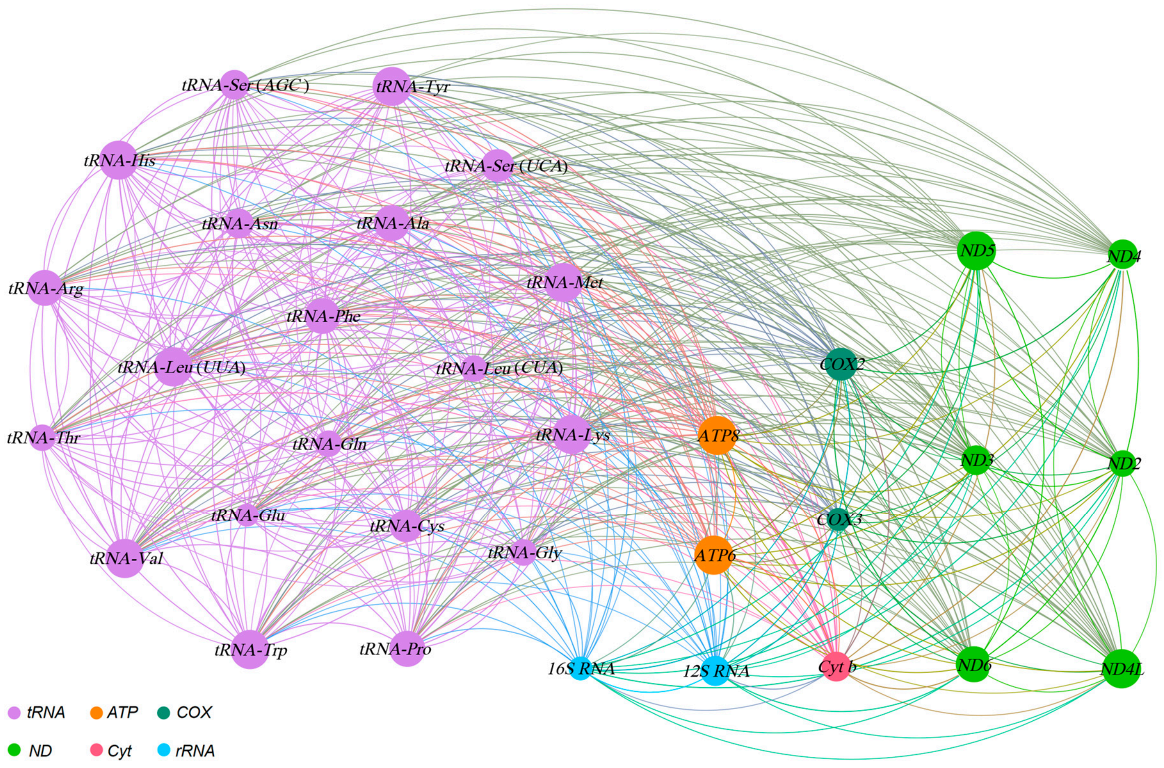

Supplementary Figure S1. The gene correlation network diagram, based on mitochondrial genome data of *G. atracaudatus*, visually represents the relationships between different genes. Using Spearman correlation analysis ($P < 0.05$, $r > 0.7$), a symbiotic network was developed in R software to explore correlations within Characidae. The network was then visualised using Gephi 0.10.1.

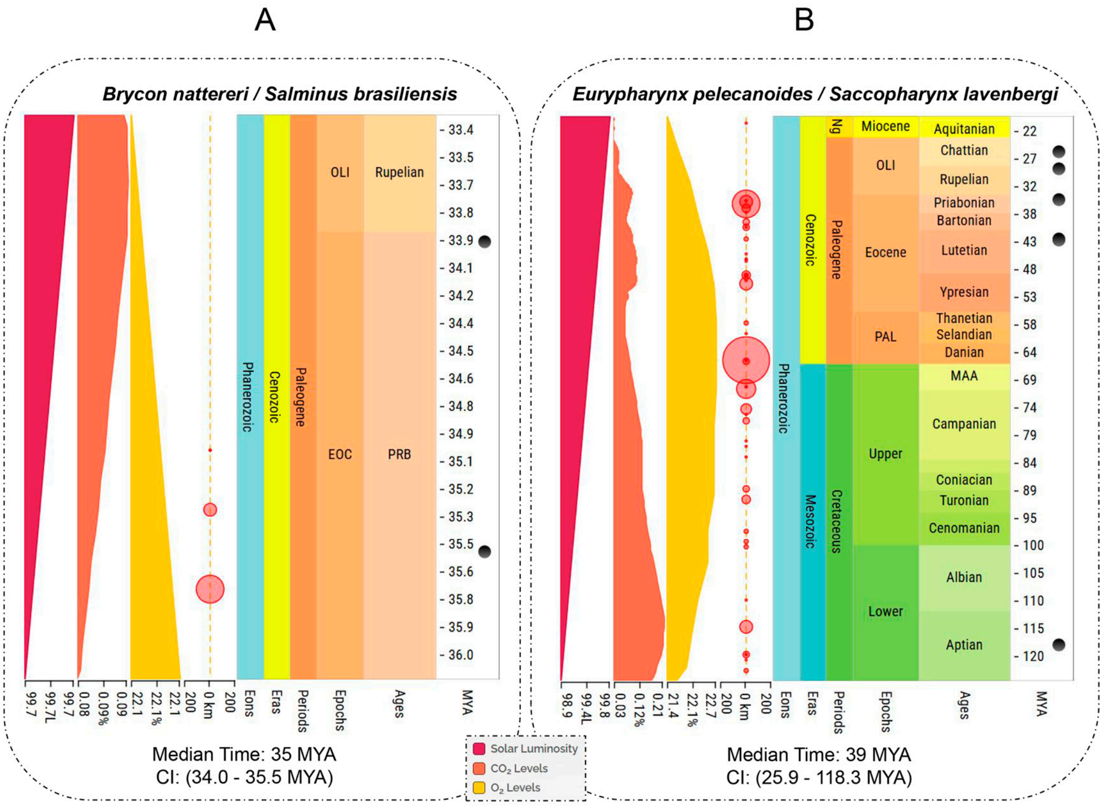

Supplementary Figure S2. Corresponding differentiation time of reference unit species.
